# The Challenges for Labour Market Policy during the COVID‐19 Pandemic*

**DOI:** 10.1111/1475-5890.12233

**Published:** 2020-07-14

**Authors:** Monica Costa Dias, Robert Joyce, Fabien Postel‐Vinay, Xiaowei Xu

**Affiliations:** ^1^ Institute for Fiscal Studies; University of Porto; ^2^ Institute for Fiscal Studies; ^3^ University College London; Institute for Fiscal Studies; ^4^ Institute for Fiscal Studies

**Keywords:** labour market policy, wage subsidies, labour demand, job vacancies, labour supply, COVID‐19 pandemic

## Abstract

The COVID‐19 pandemic is having a dramatic economic impact in most countries. In the UK, it has led to sharp falls in labour demand in many sectors of the economy and to initial acute labour shortages in other sectors. Much more than in a typical downturn, the current crisis is not simply a general slowdown in economic activity but also a radical short‐term shift in the mix of economic activities – of which an unknown, but possibly significant, amount will be persistent. The initial policy response has focused on cushioning the blow to families’ finances and allowing the majority of workers and firms to resume their original activities once the crisis subsides. These are crucial priorities. But there should also be a focus on reallocating some workers, either temporarily if working in shut‐down sectors or permanently by facilitating transitions to sectors and jobs offering better prospects and facing labour shortages. The phasing‐out of the furlough subsidies, which is projected to happen in Autumn 2020, brings this into even sharper focus since the alternative for many workers will be unemployment. Active labour market policy will need to be front and centre.

## I. A (partly?) temporary, radical restructuring of the economy

The public health response to COVID‐19 has led to sharp falls in labour demand in many sectors of economic activity in the UK. The immediate policy response to the pandemic imposed the close‐down of entire sectors of the economy, including non‐essential retail, hospitality and leisure businesses, while air travel has largely halted due to travel restrictions. These sectors alone employed over 5 million workers in the UK on the eve of the crisis, and many were left without work after the lockdown on 23 March. Add in the knock‐on effects of these closures on other industries in those sectors’ supply chains, and the proportion of the UK workforce left without productive work to do in the first few months of this crisis rose with unprecedented sharpness. Official statistics revealed that, by the end of May, over 8 million employees have lost their work at least temporarily by being furloughed. Official numbers for permanent job separations are not yet available at the time of writing, but the quick increase in claims to universal credit during April suggests that these too have spiked.

Initially, we saw significant labour shortages in other sectors. Supermarkets, warehousing and delivery services, and the NHS launched recruitment drives to cope with the coronavirus spike. Vacancies in these sectors have since fallen as these roles were filled, but they remained higher (relative to before the pandemic) than in other sectors.^1^ Technology firms such as Facebook and Google also launched recruitment drives, though partly in response to reduced hiring by start‐ups.^2^ To a far greater extent than in a typical downturn, the current crisis is not simply a general slowdown in economic activity but also a radical shift in the mix of economic activities.

Much of the restructuring of economic activities will be temporary. This points towards the need to protect firms, and employer–employee links, that have valuable long‐term futures but might otherwise struggle to make it to the other side of this crisis. The Coronavirus Job Retention Scheme was designed to achieve this (see Section II).

However, as time passes and a vaccine or cure for COVID‐19 remains elusive, it becomes ever more likely that the pandemic will have a lasting impact on the structure of the economy. Not all sectors are likely to be equally affected, but some will certainly see very persistent, or even permanent, effects that result from changes in habits, preferences and technologies. Some fraction of those now turning to online deliveries may discover they like them and will continue using them, while the rapid development and adoption of virtual technologies may inflict a sustained drop in demand for air travel and a growth in jobs that utilise, or depend on, remote working or e‐commerce. While cafes and restaurants will reopen and people will start visiting shops again, even here the recovery may be slow if consumers feel unsafe, or their economic circumstances deteriorate, or social distancing measures make such activities less enjoyable; and the balance between labour and capital may change if, for example, it is easier to comply with public health regulations using robots than humans. So while the economy after COVID‐19 will almost certainly look more similar to how it looked before the virus came to our shores than to how it looks now, the road there may be long and disruptive.

In sum, then, there is a key balance to be struck, between reallocating some workers to jobs or sectors where their future will be brighter and the need to have firms that make it through the crisis ready to quickly resume ‘business as usual’, without requiring an inefficient round of hiring of workers who will have no previous experience with that firm. Achieving that balance is not straightforward. If existing employment ties are broken now, the economy may be in a less good position to bounce back once the health crisis has passed. On the other hand, keeping workers tethered to their previous jobs risks propping up sectors that do not have a future and preventing reallocation from taking place.

## II. The initial policy response: trying to limit permanent job separations

The initial policy response focused heavily on preventing employment ties from being severed in struggling sectors (essentially, encouraging what economists call ‘labour hoarding’) and protecting the incomes of those workers. This was important for a number of reasons. Past research has highlighted the importance of ‘firm‐specific human capital’ for workers^3^ – knowledge about the company's operation and colleagues, for example, or proficiency in just the right combination of skills for the job – which means that they are able to work more productively (and hence potentially earn more) at their current firm than if they were to work somewhere else. The destruction of this ‘matching capital’ in recessions, as employment links get severed in the downturn, has been shown to derail workers’ careers, keep wages low and further dampen aggregate demand in the economy, propagating and prolonging the effects of the downturn.^4^ The costs are higher for more experienced workers, whose seniority reveals their jobs are likely a good match for their skills and whose vulnerability to the COVID‐19 infection would make it difficult for them to take alternative jobs if they lose their current ones.^5^ Moreover, even if after the crisis all workers are able to find a firm that is as well matched to them as the firm they worked for pre‐crisis, the one‐off cost of the massive round of hiring that would need to take place as the public health crisis passes would itself be significant.

In the UK, the government moved to extend loans and cash grants to firms in badly hit sectors and to subsidise up to 80 per cent of wages for furloughed workers through the Coronavirus Job Retention Scheme (CJRS). These policies insure firms against the shock, making it more likely that they will hang on to their workers through the pandemic (and their capital – something we focus on less in this paper, but which is also very important) so that business can be speedily and efficiently resumed post‐crisis. The CJRS also insures workers against falls in income, so they can continue to meet their housing costs and other spending commitments. The role of CJRS in protecting the incomes of families – and through that in preventing an even larger demand shock than that created by the requirements of social distancing, with consumers staying at home – is made especially important by the large cuts to the welfare system that have happened over the last decade.^6^

This approach was fairly well designed as an attempt to preserve the ‘matching capital’ described above, allowing workers to resume working in their original job once the crisis has passed. However, it does not address the other need we have discussed here, which is for some temporary or even permanent reallocation of workers to sectors with labour shortages and better long‐term prospects. The need for temporary reallocation to essential sectors is worth prioritising. The existence of so many furloughed workers provides an obvious source of a solution to this problem, to the extent that they have the skills required or can attain them quickly.

In the longer term, some sectors may be permanently affected – for example, aviation or non‐food retail (negatively) or healthcare (positively). The retail sector in particular was in decline long before the pandemic hit,^7^ and changes in preferences and technologies during the pandemic – with people learning how to shop online and companies investing in online platforms – mean that demand for high‐street retail is likely to decline even more rapidly going forward. By protecting firms and worker–job matches in these sectors, these policies may be delaying necessary adjustments which will be needed later. These adjustments may happen quickly once these extraordinary protective policies start being scaled back.

## III. The next phase of policy: scaling back support while promoting economic recovery

Scaling back support is likely to be a painful process for many. The very generous programmes that were put in place initially, while perhaps providing an adequate level of insurance to firms and workers against the massive shock that this pandemic represented, are not sustainable in the medium term and indeed may inhibit some efficient reallocation of resources by keeping afloat some low‐productivity firms and low‐quality worker–job matches. There are some workers – particularly those who had relatively little experience in their current firm, such as young workers – who would be better off finding new work than waiting months for their old employment to come back. It is therefore no surprise that the government started to actively seek ways of reopening the economy while providing a lighter‐touch support to firms and workers than it initially did. This is challenging because the risk of contagion remains high and the medical advances required to ensure safe social contact are still lacking. It means that both firms and workers will continue to face tough conditions, with demand predicted to remain subdued for an extended period in many sectors and with distancing requirements in the workplace affecting productivity and the organisation of work.

The next phase of policy is a gradual withdrawal of support, and this has been announced well in advance so that workers and firms can prepare for the new circumstances. In particular, the CJRS will be closed to new entrants from early July 2020 but will continue supporting firms and workers that enrolled earlier. Firms will be asked to contribute, first by paying National Insurance contributions for furloughed workers and later by sharing the cost of their pay with the government. The scheme will also allow furloughed workers to restart part‐time, by topping up pay for the proportion of work time that the workers are not doing (a measure that should probably have been part of the CJRS from the start). The subsidy of part‐time work for existing matches is similar to the short‐time subsidies that have long been a key labour market policy in some other countries^8^ – for example, France, Italy and Germany – and that have been credited for the resilience of labour markets to the worst economic shocks in these countries.

These reforms will likely be sufficiently mild to preserve many jobs of furloughed workers and keep many firms afloat in the short term. Workers whose ability to return to their full‐time position is impaired, such as parents of young children, particularly mothers facing high childcare demands, are likely to benefit the most.^9^ Moreover, the new part‐time subsidy component of CJRS is well suited to supporting a gradual restart of economic activities while avoiding crowding in the workplace, which is a key health requirement. However, the health benefits in the workplace may be counteracted by additional congestion on public transport if the policy facilitates the return of more workers to the workplace than would have happened otherwise (particularly if it leads to two part‐time workers as an alternative to one full‐time worker) and if their working schedules are not sufficiently staggered throughout the day.

**FIGURE 1:**
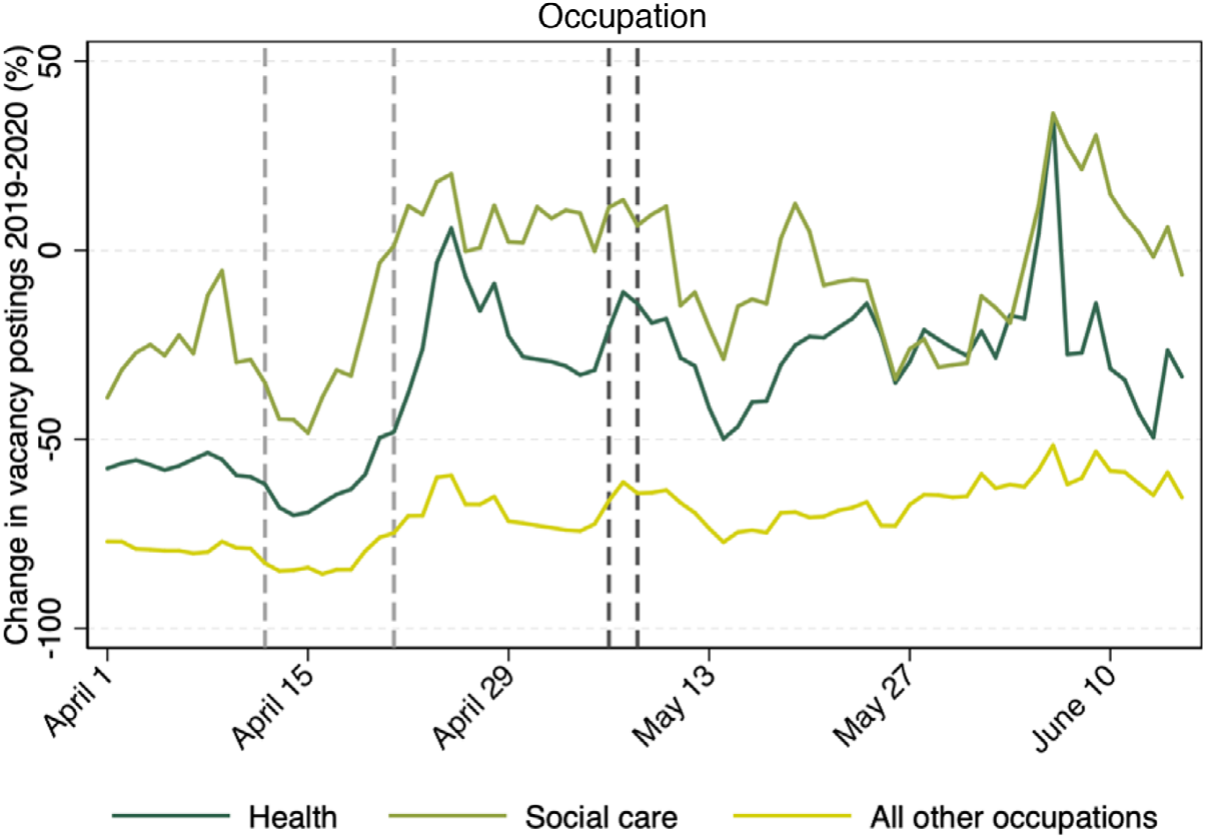
Change in new daily vacancy postings 2019 to 2020, by occupation *Note*: Trend lines show seven‐day backward‐looking moving averages. Light grey dashed lines indicate Easter Sunday (21 April 2019 and 12 April 2020) and dark grey dashed lines indicate the first May Bank Holiday (6 May 2019 and 8 May 2020). Health and social care workers are defined at the four‐digit Standard Occupational Classification (SOC) code level based on the Office for National Statistics (ONS) classification. *Source*: Costa Dias et al., 2020.

**FIGURE 2:**
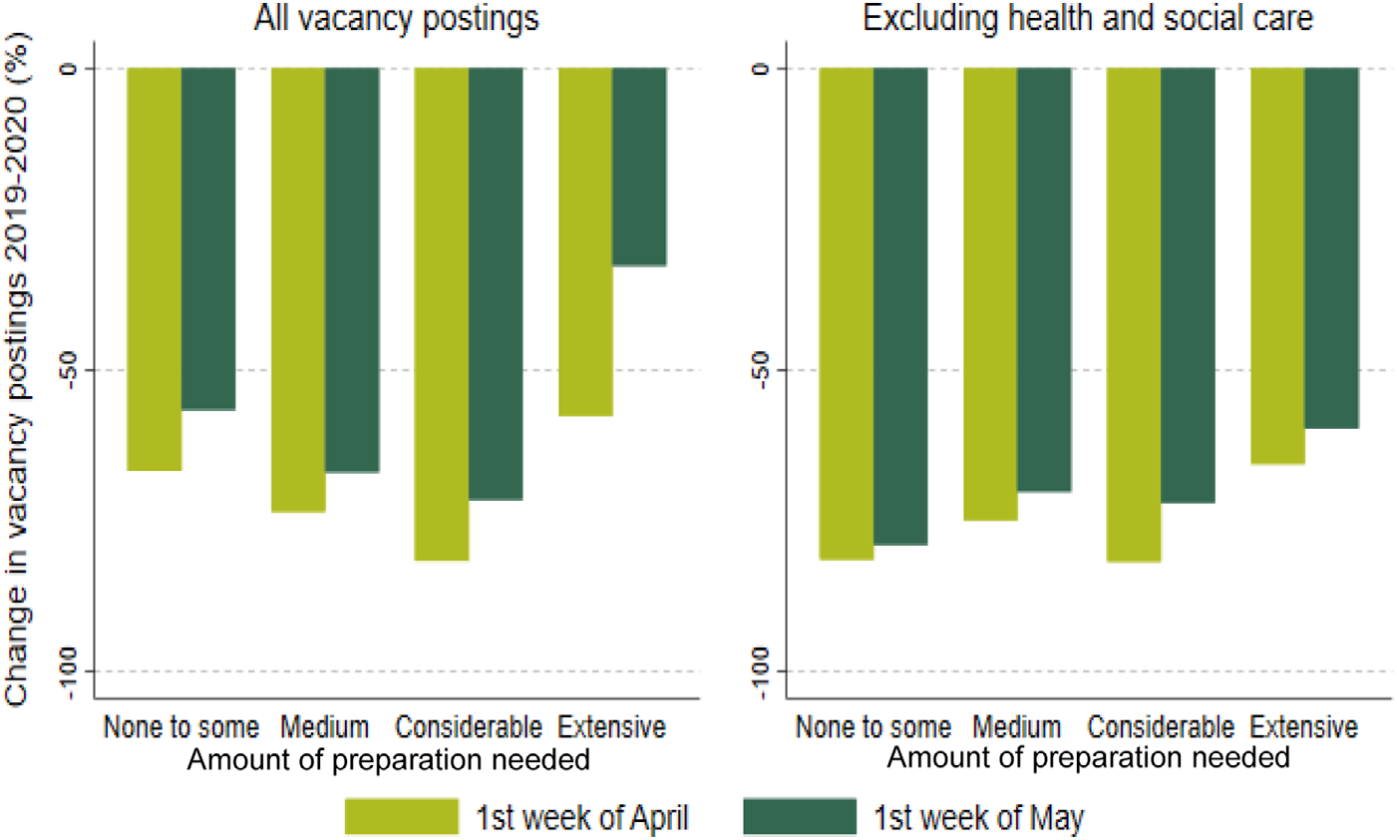
Change in new vacancy postings 2019 to 2020, by week and preparation needed *Note*: First week of April defined as Monday 1 April to Sunday 7 April in 2019 and Monday 30 March to Sunday 5 April in 2020. First week of May defined as Monday 29 April to Sunday 5 May in 2019 and Monday 27 April to Sunday 3 May in 2020. Health and social care workers defined at the four‐digit SOC code level based on the ONS classification. Job zones based on O*NET classification, mapped from US to UK SOC codes and rounded to the nearest integer. Job zones 1 (‘little or no preparation’) and 2 (‘some preparation’) are grouped together, as only 1 per cent of vacancy postings in March–May 2019 had a value of 1. *Source*: Costa Dias et al., 2020.

On the other hand, lower‐quality worker–job matches and, potentially, the most recent ones – for which there is still little information about their quality – may be severed. Likewise, firms that are experiencing especially tough conditions may face insolvency once support is scaled down. This may not be undesirable if the matches that are destroyed are those of lowest value and if the workers concerned can be quickly redeployed to more productive activities. But the latter can be difficult to achieve in the short term. Costa Dias et al. (2020) collected real‐time data on job postings to study how labour demand has been affected by the COVID‐19 pandemic. Figure 1, which is from their study, shows that the number of vacancies collapsed during April and early May 2020 compared with a similar period in 2019. The drop in the number of vacancies is especially dramatic for occupations not in health and social care, and the recovery that can be observed towards the end of April is also much more modest for these other occupations. The authors’ work also suggests that the very modest recovery in labour demand we are now seeing is not uniform across job characteristics, with, for example, a stronger demand for occupations involving close physical proximity and that are difficult to perform remotely. Whether the mix of new job vacancies is socially optimal is doubtful, as individual workers and employers are unlikely to fully internalise the infection externality or the (positive) externalities associated with innovation in technologies favouring remote work. Moreover, there is no guarantee that the skill sets of workers looking for new work will match up well with the skill requirements of new jobs. Indeed, Figure 2, also taken from Costa Dias et al. (2020), shows that recovery has been faster for occupations that require extensive training, to a large extent driven by health and social care. Other studies have shown that it is low‐skilled workers who have been most at risk of losing their jobs either temporarily or permanently.^10^ Government intervention is therefore likely to be warranted.

## IV. Policy going forward: facilitating the reallocation of workers

As argued above, policies currently in place are designed to preserve aggregate matching capital by maintaining the employer–employee link in matches that have been made temporarily unprofitable by the lockdown but that are likely to quickly recover much or all of their value after the crisis. Going forward, policy must pursue another objective: that of getting workers into the right jobs. This means helping displaced workers and workers whose jobs have become durably unproductive to find alternative, high‐value employment.

### How might we achieve this in practice?

Without intervention, labour reallocation to socially desirable activities will happen very slowly if labour demand remains as subdued as we report above. It seems likely that some employers are being especially cautious and abstaining from hiring altogether given the unprecedented levels of uncertainty^11^ and the increasingly clear understanding that the recovery from this crisis will be longer and more volatile than predicted at the start of the lockdown. One way the government can reduce uncertainty and stimulate labour demand is to implement policies specifically designed to protect workers and firms against major shocks and to credibly commit to them in the longer term. For instance, one feature that sets the Coronavirus Job Retention Scheme apart from short‐time work subsidies operating elsewhere is that the UK scheme is specifically designed to be a temporary measure and will be withdrawn gradually as jobs resume or are closed down. Specifically, the current policy fails to protect firms and workers that are not in the scheme by July 2020. In contrast, other countries – such as Germany, Italy and France – use short‐time subsidies as part of a permanent set of policies designed to stabilise the labour market through economic fluctuations. If the pandemic continues shaking up the economy with future shock waves, employers in Germany know that their firms and workers will be protected by short‐time subsidies. In contrast, those in the UK can only hope for new protections to be legislated, and know this may only happen if a sufficiently large proportion of the economy is affected. A more permanent version of the CJRS could not only prevent job separations down the line but also help to provide the insurance needed to motivate some employers to start hiring, hence alleviating some of the current tensions in the labour market.

A number of other policy tools can be used together to promote job creation, facilitate labour reallocation and protect workers and their families against economic deprivation. A direct approach is to take advantage of the low opportunity cost of public sector hiring to launch public investment projects that will also provide work experience and skills development for the unemployed. In addition, wage subsidies (other than the CJRS), hiring subsidies, and benefits and job search assistance for the unemployed can also be used to facilitate the reallocation of labour and insure families. The efficient implementation of those policies will be very difficult, as it will have to factor in many variables simultaneously, with little data to inform choices.

Wage and hiring subsidies will have to be varied along dimensions not traditionally considered by economists or policymakers, such as ease of working from home, physical proximity in the workplace or vulnerability to the virus. For example, the reallocation of young workers to socially desirable but risky occupations should be incentivised, while older workers should stay in safe occupations. Moreover, as with any subsidy scheme, wage and hiring subsidies are fraught with moral hazard issues. Those issues have so far, and probably rightly, been considered of secondary importance, but they are more than likely to gain importance as the economy recovers.

Job search assistance will probably be key: recent research has shown that younger workers are the ones most likely to have lost their job during the crisis^12^ and to be suffering from the well‐documented scarring effects of job loss, which are known to be especially strong in recessions.^13^ Moreover, while the CJRS currently covers 80 per cent of the income of furloughed workers, those who have been laid off have suffered much worse income losses. These facts mean that a policy aiming to facilitate labour market reallocation cannot be dissociated from policies supporting the unemployed. Job search assistance measures are widely considered a cost‐effective way of reducing unemployment duration^14^ and should be considered very seriously.

For these types of policies to be successful, it is crucial to correctly identify possible ‘good matches’ between vacant jobs and job seekers. To that end, it will be necessary to monitor the characteristics (in terms of skill requirements, geography, etc.) of new job openings in as close to real time as possible, to keep track of what and where the needs of the economy are.

However, good matches will be difficult to make if there are systematic mismatches between the skills that unemployed workers have and the demands of the jobs being created. This is likely to be the case now, as the jobs where vacancies have held up during the lockdown tend to be highly skilled while low‐skilled workers have been disproportionately affected by temporary and permanent job losses. Training in the workplace can be an important part of the solution. Past research has cast doubt on the value of publicly provided professional training,^15^ but more recent evidence shows that some workers take substantial amounts of valuable training in their jobs.^16^ Finding ways to further incentivise the right forms of training – for instance, through apprenticeships targeted at young workers and delivered in work – could help in closing the gap between the workers’ skills and the requirements of new jobs. The government may take a forward‐looking approach and equip people with the skills that are likely to be in demand in the future, given a potential shift towards e‐commerce and the need to move to a net‐zero economy.

## V. Conclusion

The nature of the economic shock associated with COVID‐19 is highly unusual. Not only is the scale of the downturn likely to be large; the types of economic activity that we are doing in the UK have changed radically in a matter of days. Much of that change is likely to be temporary, and policy has so far – and rightly – focused on keeping the economy ready to return to ‘business as usual’ as seamlessly as possible once the crisis subsides. But beyond the immediate, dramatic impact of the lockdown, there is emerging evidence that employers are expecting large changes to their workforce over the next year or so.

Over the coming months, policymakers will face a very delicate balancing act between tiding over those employer–employee matches that are likely to recover their value once the crisis subsides, and getting workers who have lost their jobs, or whose jobs have become permanently unviable, not only back into work but also back into the right jobs. This paper has highlighted some of the main challenges that come with that difficult task.

## Footnotes

1 Costa Dias et al., 2020.

2
https://www.businessinsider.com/tech-giants-hiring-job-openings-coronavirus-apple-google-amazon-facebook-2020-4?r=US%26IR=T.

3 Jacobson, LaLonde and Sullivan, 1993; Davis and von Wachter, 2011; Fujita and Moscarini, 2017.

4 Moscarini and Postel‐Vinay, 2016 and 2019.

5 Fujita, Moscarini and Postel‐Vinay, 2020.

6 Blundell et al., 2020.

7 Gardiner and Tomlinson, 2019.

8 Cahuc, Kramarz and Nevoux, 2018; Giupponi and Landais, 2018; Adams‐Prassl et al., 2020.

9 Adam, 2020.

10 Adams‐Prassl et al., 2020; Andrew et al., 2020.

11 Baker et al., 2020.

12 Benzeval et al., 2020.

13 Schmieder, von Wachter and Heining, 2019.

14 Rosholm, 2014.

15 Card, Kluve and Weber, 2010.

16 Blundell et al., 2019.
